# Divergent Evolution of Male Aggressive Behaviour: Another Reproductive Isolation Barrier in Extremophile Poeciliid Fishes?

**DOI:** 10.1155/2012/148745

**Published:** 2011-10-23

**Authors:** David Bierbach, Moritz Klein, Vanessa Saßmannshausen, Ingo Schlupp, Rüdiger Riesch, Jakob Parzefall, Martin Plath

**Affiliations:** ^1^Department of Ecology and Evolution, J.W. Goethe University Frankfurt, Siesmayerstrasse 70-72, D-60054 Frankfurt am Main, Germany; ^2^Department of Zoology, University of Oklahoma, 730 Van Vleet Oval, Norman, OK 73019, USA; ^3^Department of Biology & W. M. Keck Center for Behavioral Biology, North Carolina State University, 127 David Clark Labs, Raleigh, NC 27695-7617, USA; ^4^Department of Ethology, University of Hamburg, Martin-Luther-King-Platz 3, 20146 Hamburg, Germany

## Abstract

Reproductive isolation among locally adapted populations may arise when immigrants from foreign habitats are selected against via natural or (inter-)sexual selection (female mate choice). We asked whether also intrasexual selection through male-male competition could promote reproductive isolation among populations of poeciliid fishes that are locally adapted to extreme environmental conditions [i.e., darkness in caves and/or toxic hydrogen sulphide (H_2_S)]. We found strongly reduced aggressiveness in extremophile *P. oecilia mexicana*, and darkness was the best predictor for the evolutionary reduction of aggressiveness, especially when combined with presence of H_2_S. We demonstrate that reduced aggression directly translates into migrant males being inferior when paired with males from non-sulphidic surface habitats. By contrast, the phylogenetically old sulphur endemic *P. sulphuraria* from another sulphide spring area showed no overall reduced aggressiveness, possibly indicating evolved mechanisms to better cope with H_2_S.

## 1. Introduction

### 1.1. Ecological Speciation

Divergent natural selection has the potential to drive adaptive trait divergence along environmental gradients [1], but can also lead to the evolution of reproductive isolating barriers [2, 3]. During ecological speciation, reproductive isolation results from ecologically based divergent selection, and prezygotic isolation may arise as a byproduct of local adaptation if immigrants from ecologically divergent habitats are selected against [3]. This can be owing to natural selection, if immigrants show reduced viability [4–6], or sexual selection, if poorly adapted individuals have a disadvantage in mate competition [5, 7, 8]. Furthermore, ecological speciation may also be driven by selection against hybrids with intermediate phenotypes [9], behavioural isolation based on a “magic trait” [10–14], and sensory drive [15].

Our present paper briefly collates our current knowledge regarding trait divergence and especially mechanisms of reproductive isolation among different locally adapted populations of livebearing fishes (Poeciliidae), currently undergoing ecological speciation processes in response to “extreme” conditions (see below). Using both lab-reared as well as wild-caught fish we then demonstrate that divergent evolution of male competitive abilities (aggressive behaviour) in extremophile fishes may play yet another role in maintaining reproductive isolation among different locally adapted populations: adaptation to extreme habitat conditions appears to have selected for reduced aggressiveness, and we show that this renders potential migrant males from extreme habitats less competitive in intrasexual combat when the resident males inhabiting benign habitats show “normal” aggressive behaviour. As the mating system of our study species is based on male dominance hierarchies, with dominant males aggressively defending small shoals of females from intruders [16–18], we argue that this pattern directly translates into reproductive inferiority of such migrant males.

### 1.2. Life in Extreme Habitats

Habitats can be considered extreme if certain characteristics of the environment are outside of the range normally experienced by a species and if organisms colonizing this particular habitat type experience an initial reduction in fitness [19, 20]. For example, some extreme environments are characterized by exceptionally high concentrations of hydrogen sulphide (H_2_S): deep-sea hydrothermal vents, hydrocarbon seeps, as well as intertidal zones, salt marshes, mudflats, and sewage outfalls, where H_2_S is usually of biogenic origin [21–24]. H_2_S inhibits aerobic respiration due to its interference with mitochondrial respiration and blood oxygen transport, but also leads to extreme hypoxia in the water [21, 22]. This makes H_2_S acutely toxic to most metazoans even in micromolar amounts, and accordingly, pulses of H_2_S discharge have been reported to be the source of mass mortalities [22].

An environmental toxicant like hydrogen sulphide that requires energetically costly behavioural (i.e., actively avoiding microhabitats with high levels of toxicity) and physiological adaptations (various forms of detoxification) by animals exposed to it will certainly have a profound influence on the evolutionary trajectories of populations experiencing the stressor [6]. For instance, when exposed to H_2_S and hypoxia, livebearing fishes resort to aquatic surface respiration (ASR) and, thus, exploit the more oxygen-rich air-water interface [25]. Under experimental conditions, Atlantic mollies (*Poecilia mexicana*) have been shown to spend more than 60% of their time performing ASR when exposed to sulphidic water [25], and in natural populations *P. mexicana *have been observed to spend up to 84% of their time performing ASR [26]. However, while access to the water surface (i.e., the possibility to perform ASR) is a strong predictor of short-term survival in fish exposed to H_2_S-containing water [25], time spent at the water surface clearly trades off against the time fish can spend foraging. Hence, fish from H_2_S-containing habitats tend to have less food in their guts and lower body condition than conspecifics from nonsulphidic habitats [26–29].

Beside toxicants, perpetual darkness, like in cave ecosystems, can represent an extreme condition for typical surface-dwelling organisms like *P. mexicana *[30]. Darkness renders visual orientation and navigation an impossible task, and cave organisms need to develop specific adaptations to cope with this situation [31–34]. Cave animals (especially crustaceans and fishes) are widely used model organisms to study the evolutionary effects of permanent darkness on various traits, including improved nonvisual sensory systems and increased starvation tolerance (e.g., [34–39]).

### 1.3. Ecological Speciation in Extremophile Poeciliid Fishes

Notwithstanding all the adverse effects of H_2_S, several species of livebearing fishes (Poeciliidae) have been documented to thrive (and speciate) in waters containing exorbitant concentrations of H_2_S. Among them are sulphur endemics like the sulphur molly (*Poecilia sulphuraria*) and widemouth gambusia (*Gambusia eurystoma*) [8, 27, 40], as well as species that are currently undergoing ecological speciation, like certain populations of *P. mexicana* [5, 6, 28, 41–43].

Of particular interest are different locally adapted *P. mexicana* populations in the Cueva del Azufre system (Tabasco, Mexico), a system that is characterized by the simultaneous action of two strong selective forces: permanent darkness in subterranean parts of streams and toxic H_2_S [6, 30, 44, 45] of volcanic origin [46–48]. Within a small geographic range of only few kilometres, reproductively isolated populations of *P. mexicana* inhabit environments characterized by all possible combinations of these two factors: a toxic cave (Cueva del Azufre, CA), a nontoxic cave (Cueva Luna Azufre, LA), and toxic surface waters; however, a small cascade separates all extreme habitats from nonsulphidic, normoxic sites (for discussion see [42]).

Another system considered in our present study is the sulphur molly system situated at the Baños del Azufre near Teapa (Tabasco, Mexico). This system is characterized by even higher H_2_S concentrations (around 230 *μ*M [8, 43]). Just like in the Cueva del Azufre system, no barriers other than presence of environmental stressors prevent movement of fish among different habitat types in this system [43]. *P. sulphuraria* forms a monophyletic sister clade with phylogenetic affinity to a northern clade of *P. mexicana* rather than *P. mexicana* inhabiting the clear-water habitats in the vicinity of the Baños del Azufre [6]. Thus, sulphur mollies appear to represent a phylogenetically old sulphur-adapted lineage and have been considered a potential “endpoint” of H_2_S adaptation [27].

Extremophile *P. mexicana* in the Cueva del Azufre system are characterized by site-specific local adaptations in several behavioural (e.g., [25, 26, 49–51]), dietary [52], female and male life-history [27–29, 53, 54], morphological [17, 43, 45, 55, 56], and physiological traits [17, 57], and there is strong evidence for convergent patterns of H_2_S adaptations across both aforementioned sulphur systems [6, 27].

### 1.4. Reproductive Isolating Barriers in Extremophile Poeciliids

Gene flow between populations with different ecological backgrounds in the Cueva del Azufre system is virtually absent with the exception of some degree of genetically detectible migrants from CA found outside of that cave (inside the El Azufre River, EA; [41, 42]). This may be due, in part, to the release of Barbasco, a fish toxicant containing rotenone, during an annual fertility ceremony (La Pesca) of the indigenous Zoque people inside the CA. As Barbasco does not lead to 100% mortality rates, it was suggested that gene flow between the two habitat types may actually be mediated by a certain degree of downstream drift of sedated individuals [58].

Strong reproductive isolation among populations from ecologically divergent habitat types appears to be the result of a combination of natural selection (i.e., direct effects of toxicity, darkness, and predation) and sexual selection through female choice [5, 8, 26, 59], both of which are acting against immigrant individuals. Specifically, H_2_S was shown to be a strong selective force in the aforementioned systems as revealed by reciprocal translocation experiments between nonsulphidic and sulphidic surface habitats [5]. Fish from nonsulphidic habitats had low survival in sulphidic habitats, whereas fish from sulphidic habitats performed poorly under nonsulphidic conditions. Those differences are underlined by tests on H_2_S tolerances as fish from sulphidic habitats exhibited consistently higher tolerances than fish from nonsulphidic habitats [6]. The high mortalities of fish in translocations from sulphidic into nonsulphidic environments were hypothesized to be caused by oxidative stress, as oxygen is inherently toxic due to its biotransformation into reactive oxygen species, and organisms have evolved biochemical pathways with antioxidant activity (e.g., superoxide dismutase, catalase, and glutathione systems [60]). During hypoxia, the expression of antioxidant enzymes is often downregulated [61, 62], such that subsequent exposure to normoxic conditions causes substantial oxidative stress with profound fitness consequences [61, 63]. Oxidative stress, possibly in combination with the often poor body condition and energy limitation of fish from sulphidic habitats [25, 27–29, 52], may explain the high mortality seen in migrants from sulphidic to sulphide-free environments.

Contrary to translocations between sulphidic and nonsulphidic habitats, a transfer of fish between sulphidic cave and surface habitats had no effect on survival in either direction. This is not unexpected, as presence or absence of light is unlikely to affect survival within only 24 h. Nevertheless, common garden experiments found that while surface females fail to reproduce in darkness, cave females reared in light are not affected [59]. This is congruent with the aforementioned pattern of unidirectional gene flow from the inside of the caves towards the outside in the Cueva del Azufre system [6, 41, 42]. A further natural selection factor against immigrants was uncovered through similar translocation experiments (outside versus inside cave) that involved the presence of a predator (a giant water bug of the genus *Belostoma*) as heteropterans were more likely to attack cavefish in light but surface fish within the cave [64].

Beside environmental factors acting more or less directly on the viability of migrants in foreign habitats also sexual selection was found to constitute a reproductive isolation barrier. Thus far, only effects of intersexual selection (female mate choice) were assessed. For example, females from the Cueva del Azufre system, including normal surface habitats, sulphidic surface habitats (EA), and the Cueva del Azufre cave (front chambers of CA, which still receive some dim light), discriminate against males from foreign habitats and preferentially associate with males from their own habitat type [5]. Similarly, in the sulphur molly system female *P. mexicana* show strong assortative mating under nonsulphidic conditions, that is, associated less with males of the sulphur-endemic *P. sulphuraria* [8]. Immigrant males from ecologically divergent habitats are consequently at a disadvantage by sexual selection (see also [65, 66]).

In the present paper, we addressed another aspect of sexual selection, namely, intrasexual selection, and asked whether divergent evolution of male aggressive behaviour (i.e., competitive abilities) could play another role in facilitating reproductive isolation among diverging populations by selecting against (maladapted) migrant males. Cave mollies from CA are well known for their reduced aggressiveness [16, 17], and this reduction appears to increase gradually from the entrance to the innermost parts of the cave [67, 68]. When analysing aggressive behaviour with light of various intensities fights occurred at first at 5 lux [69]. When hybrids and backcrosses between cave and epigean fish were tested [68], the frequency of distribution patterns for aggressive fin erection and S-position revealed a genetically based reduction of the aggressive behaviour within CA fish. The F_1_ generation had an intermediate value for the average, and the variability was practically halved in comparison to the epigean forms. It was concluded that the reduction for aggression is based and controlled by a polygenic genetic system. Furthermore, it was suggested that costly aggressive behaviours lack stabilising selection in darkness where visual perception of an opponent is prohibited; accordingly, reduced aggression was interpreted as a consequence of cave adaptation, that is, evolution under perpetual darkness [17]. Due to the young age of the CA cave molly this reduction process is thought to be still ongoing, eventually leading to the complete reduction of aggressive behaviour in this cave-dwelling population. Despite the extensive work on male aggression in fish from the CA, nothing is known about male aggressive behaviour of *P. mexicana* from the newly discovered sulphide-free Cueva Luna Azufre (LA) which is thought to have been colonized even more recently than the neighbouring CA cave [45]. Moreover, little is known about whether or not presence of toxic H_2_S also plays a role for the reduction of aggressive behaviour and, if this was the case, whether evidence for convergent evolution in other drainages containing H_2_S can be uncovered. Our hypothesis that not only darkness in caves, but also H_2_S might affect the evolution of aggressive behaviour is based on the following considerations. Fish from H_2_S-containing waters were found to have lower body conditions and fat stores [27–30, 52], most probably due to altered time budgets because of the amount of time being spent in ASR [26] and the physiological cost of H_2_S detoxification [22]. These factors have been hypothesized to account for the observed heritable reduction of male sexual activity and sexual harassment of females found in all extremophile populations [50, 70].

In the present study, we asked the following specific questions.

- What are the independent and interactive effects of H_2_S and darkness on the evolution of aggressive behaviour in the Cueva del Azufre system? Do both stressors (H_2_S and darkness) select for reduced aggression? We observed the outcome of dyadic aggressive interactions in male pairs from all divergent populations in the Cueva del Azufre system. For *P. mexicana* ecotypes from this system broad-sense heritability of population differences in the tendency to respond aggressively could be estimated by investigating laboratory- (i.e., common garden-) reared fish.
- Is there evidence for convergent evolution (i.e., reduction) of aggressive behaviour in another system with high and sustained H_2_S, namely, *P. sulphuraria* inhabiting the Baños del Azufre? While fish from the Cueva del Azufre system are easy to maintain and readily reproduce in the laboratory under nonsulphidic light conditions [17, 59], none of our attempts to breed *P. sulphuraria* have been successful so far, as fish would typically die within some weeks upon transfer to the lab. Therefore, for the comparisons among ecotypes in this system we had to rely on wild-caught fish and conducted our experiments on site in Southern Mexico.
- Cave-adapted blind characids (*Astyanax mexicanus*) show reduced aggression [71], but were found to increase aggressiveness and to defend small feeding territories when starved [72]. Based on these findings, we asked if *P. mexicana* from CA and EA (i.e., populations showing reduced aggression) would also become more aggressive when starved and thus compared aggressive behaviour of male dyads that had undergone different feeding treatments (high diet versus one week starvation).
- Does divergent evolution of aggressive behaviour in extremophile mollies translate into males being inferior in competition with more aggressive males from populations evolving under benign conditions? We simulated a potential migration scenario where the least aggressive CA males were paired with males from a nonsulphidic, normoxic surface stream and investigated their aggressive interactions as well.

## 2. Materials and Methods

### 2.1. Study System

The Atlantic molly,* P. mexicana,* is widespread in freshwater surface habitats along the Atlantic versant of Central America [73]. For our experiments we used both wild-caught fish (experiment 3) and lab-reared descendents of wild-caught fish (all other experiments). Laboratory-reared *P. mexicana* originated from the Río Oxolotán (Ox), a river with mostly clear water in the vicinity of the caves [6, 30], and from the brackish coastal waters near Tampico (Tam; Tamaulipas, eastern Mexico). As representatives from extreme habitats we used descendents from the sulfidic El Azufre (EA), a creek flowing out of the Cueva del Azufre [6, 30]. We furthermore used fish from three distinct cave chambers of the sulphidic Cueva del Azufre (chamber II (CA-II), chamber V (CA-V), and chamber X (CA-X); after [44]) and males from the newly discovered nonsulphidic Luna Azufre cave (LA, [45]). Wild-caught fish for experiment  3 were *P. mexicana* from the nonsulphidic Río Ixtapangajoya (IX, [74]) and from chamber II of the Cueva del Azufre (CA-II), as well as male *P. sulphuraria* (PS) from the Baños del Azufre [40]. GPS coordinates for all sampling localities are given in Table 1.

### 2.2. Test Fish and Their Maintenance

Laboratory stocks were maintained in large, randomly outbred single-species tanks at the Department of Ecology and Evolution of the University of Frankfurt or at the Department of Zoology at the University of Oklahoma in Norman. At both facilities, fish were reared as mixed-sex stocks in 200-L (Frankfurt: Tam, Ox, LA) or 1,000-L tanks (Norman: EA, CA-II, CA-V, CA-X) at 25–27°C under a 12 : 12 hrs light : dark cycle (Frankfurt) or ambient light conditions (Norman) and were fed *ad libitum* at least once daily with commercial flake food. All lab-reared fish were kept under normoxic conditions without H_2_S, and test fish were descendants of wild-caught fish of the 2nd to 4th laboratory generation.

In experiment  3 we used wild-caught fish, because *P. sulphuraria* could not be maintained under laboratory conditions for more than some weeks, most probably due to their high degree of adaptation to H_2_S-containing water [6]. Upon capture, fish were transferred into closed and aerated (38 L, 43 × 31 × 32 cm) black Sterilite containers, and we gave them 24 h to acclimate before testing them in a field laboratory as described below.

### 2.3. Behavioural Tests

#### 2.3.1. General Testing Procedure

We determined male aggressive behaviours during dyadic encounters by analysing contests staged between pairs of males in a small test tank measuring 30 × 20 ×20 cm. To avoid any confounding effects of previously established dominance and/or familiarity (see [75, 76]), males of each dyad were taken from different stock tanks. We separated both males by an opaque filter sponge while all sides of the test tank were taped with grey paper to minimize disturbances from the outside. The bottom of the tank was filled with black gravel, and water was kept at 27–29°C and aerated. All experiments were performed with normoxic, nonsulphidic water. Males could habituate to the test tank overnight, and fight observations took place the next day between 09:00 and 13:00. As even size-matched males differed slightly in their fin and general body colouration and were thus easily distinguishable, we noted individual characteristics of both males prior to the fights. At the start of the experiment, the partition separating both males was lifted, and we noted male-male interactions for a maximum of 10 minutes, starting with the first male-male interaction. We focused on three aggressive behaviours that occur frequently in *Poecilia* spp. (after [16]). (1) S-position: this threatening behaviour usually initiates a fight. Males swim in a parallel or antiparallel position and bend their bodies in an S-shaped manner while all unpaired fins are erected. (2) Tail beats: S-positons are often followed or superimposed by tail beats, which are fast moves of head and tail in opposing direction that either touch the opponent's body or send shock waves towards the opponent. (3) Bites: we defined all incidences of ramming and mouth attacks into the direction of the opponent as bites, because these behaviours occur too fast and are too similar to be distinguishable by the human eye.

We also recorded the duration of the fights until dominance was established. Contest outcome was indicated by behavioural differences between the competitors. Folded fins, head-down posture, and a position at the periphery of the tank typically characterized the loser of the contest [77]. Winners, on the other hand, constantly chased and further attacked the losers with spread fins while occasionally performing S-positions. We, therefore, separated both males immediately after dominance was established to avoid serious injuries. If no dominance was established after 10 minutes of observation we terminated the fight and scored fights as “no clear winner”; those trials were discarded from the analysis of fighting durations, while fight durations were scored as “0” when no aggressive behaviour occurred at all. After a contest, body size of all males was measured as standard length (SL) to the nearest millimetre by laying the fish flat on plastic foil-covered millimetre paper, and we transferred the males back into their respective stock tanks. Despite the loss of single scales, no severe injuries and no mortality related to the experiments were observed.

#### 2.3.2. Experiment  1: When Do Fights Escalate?

The assessment of an opponent's resource holding potential (RHP; see [78]) is crucially connected to the opponents' body size difference in poeciliid fishes [77], and at least in swordtail fish (*Xiphophorus *spp.) fighting intensity (determined as numbers of bites per minute) correlates negatively with the opponents' size difference, but simultaneously was also found to vary greatly when size differences were small [77]. We, therefore, first examined the correlation between fight intensity (determined as numbers of bites per minute) and the opponents' absolute body size (measured as standard length) difference using Spearman's rank order test. We tested 17 male dyads of *P. mexicana* (Tam), while relative size differences within each pair ranged from 0% to 47% (mean pair size: 35.8 ± 1.8 mm). Fight intensity was plotted against opponents' absolute body size difference, and a logarithmic model was used to generate a reference line.

Despite the predicted large degree of variability in fight intensity (see [77]), escalating fights in swordtails (defined as both males biting each other) are more often found when body size differences are small [76, 77]. As the aim of our study was to compare maximum aggressiveness of escalating fights in different *P. mexicana* populations we furthermore tried to estimate the maximum relative opponents' size difference (determined as fraction of standard length the larger male exceeded the smaller male) up to which escalating fights can be observed. To do so, a score expressing how equally both males dedicated their aggressions towards each other in a dyad was calculated as a measure of escalation as: 1 – Abs((sum aggressive behaviours male one − sum aggressive behaviours male two)/sum of aggressive behaviours of male one and male two).

Escalation scores could range between 0 and 1, with values around 0 indicating that only one male showed aggressive behaviour (uneven, no escalated fight) and values around 1 indicating that both males dedicated similar amounts of aggressive behaviours towards each other (even, escalated fight). Scores were plotted against arcsine- (squareroot-) transformed relative body-size differences, and a logistic 4-parameter curve estimation (upper constraint set to 1, lower constraint to 0) was used to determine the Evenness_50_-score (body size difference at which the score value is 0.5). When opponents' body size differences exceeded the body size difference at the Evenness_50_-score we assumed fights to be less escalated.

For statistical reasons, scores and body-size differences equal 0 were substituted by 0.001, as logistic models require positive nonzero data.

#### 2.3.3. Experiment  2: Evolution of Male Aggressiveness in Response to Environmental Stressors

It was our intention to disentangle the relative effects of sulphur and darkness on the evolution of aggressive behaviour. In a first step we tested whether populations from sites with the same combination of ecological stressors would show comparable levels of aggressive behaviour and, thus, compared the two populations from nonsulphidic surface sites (Tam and Ox) as well as the three CA populations (CA-II, CA-V, CA-X) using similar MANCOVA and ANCOVA models as outlined below. The MANCOVA models with numbers of aggressive behaviours per fight as dependent variables neither detected a significant population difference between both nonsulphidic surface populations (*F*_3,20_ = 0.44; *P* = 0.77) nor between the three CA populations (*F*_6,38_ = 1.11, *P* = 0.38). When comparing fight durations using ANCOVA models we did not find population differences as well (surface: *F*_1,22_ = 1.86, *P* = 0.19; CA: *F*_2,20_ = 0.26, *P* = 0.77).

Based on these results we analysed numbers of aggressive behaviours per fight in seven populations of *P. mexicana* from different ecological backgrounds in our main analysis (see Table 1 for a detailed description of collection sites): A MANCOVA model with “number of S-positions,” “number of tail-beats,” and “number of bites” as dependent variables and “sulphur” (present/absent) as well as “light” (present/absent) as fixed factors was employed, and “mean pair size” as well as “body size difference” (arcsine (square root) transformed) were included as covariates. We initially included all levels of interaction terms between both main factors and both covariates, but removed interactions from the final model as none of them were significant (not shown). Prior to all analyses, all dependent variables were log transformed and afterwards checked for normal distribution by Kolmogorov-Smirnov tests.

Fighting durations were analysed in a separate ANCOVA model with “sulphur” (present/absent) as well as “light” (present/absent) as fixed factors and “mean pair size” as well as “body size difference” as covariates. No interaction term was significant (not shown), and thus interactions were excluded from the final model.

#### 2.3.4. Experiment  3: Aggressiveness in the Sulphur Endemic *P. sulphuraria*

In this experiment we compared male aggressive interactions among wild-caught individuals of the sulphur endemic *P. sulphuraria *(PS; *N* = 9) and two populations of *P. mexicana, *one from a freshwater surface habitat, the Río Ixtapangajoya (IX; *N* = 7), the other one from the sulphidic Cueva del Azufre (CA-II; *N* = 7 male dyads), in a field laboratory. We used small mice cages (23 × 15 × 16.5 cm) instead of our standard test tanks and separated males overnight by opaque plastic sheets. As described for experiment  2, we analysed aggressive behaviours among those three populations through MANCOVA with “mean pair size” as well as “body size difference” as covariates and fight durations in an ANCOVA with “mean pair size” as well as “body size difference” as covariates. In both analysis, interaction terms between the main factor “population” and the covariates were initially included, but removed from the final model as neither had a significant effect.

We used Fisher's LSD tests for pairwise *post hoc* comparison of overall levels of aggression (mean sum of all aggressive interactions per fight) as well as fight durations among populations. In addition, we also compared all three different kinds of aggressive behaviours separately by use of one-way ANOVA and applied Fisher's LSD tests to identify the source of variation when a significant population effect was detected.

#### 2.3.5. Experiment  4: Aggressiveness and Food Limitation

As food limitation is predicted to influence the occurrence of aggressive behaviours in fish [79], we compared the intensity of male fights under normal food supply (daily *ad libitum *feeding regime) with fights of males that were subject to a 1-week starvation period. To do so, we separated males from the CA-X and EA populations for 6 days in 50-L tanks and deprived them of food. After this period males that had not been fighting against each other (i.e., stemmed from different tanks) were transferred into our standard test tanks, and fights were observed on the following morning (hence, males starved for 7 days altogether). We analysed numbers of aggressive behaviours per fight (including data for nonstarved males from experiment  2) in a MANCOVA model with “population” (2 levels) and “treatment” (non-starved/starved) while including “mean pair size” as well as “body size difference” as covariates. Analogously, fight durations were analysed with the same factors and covariates in an ANCOVA model. In both analysis, interaction terms of the main factors “population” and “treatment” and the covariates were initially included but removed from the final model as neither had a significant effect.

#### 2.3.6. Experiment  5: Male Aggression as Reproductive Isolation Barrier

Reproductive isolation is crucial for speciation processes, and intrasexual competition may provide one possible mechanism to exclude immigrants from reproducing in foreign habitats. To test this idea, we staged contests between size-matched males from CA (CA-X; mean SL = 30.4 ± 0.7 mm) versus males from a sulphide-free surface habitats (Ox population; 30.7 ± 0.8; paired *t*-test on size differences: *t*_12_ = −0.81; *P* = 0.45). Chi² tests were employed to compare numbers of fights won by males from either population, and numbers of aggressive behaviours shown by the two ecotypes within each male dyad were analysed using paired *t*-tests. We further recorded and compared all sexual behaviours (nipping and copulation attempts, so-called thrusting; see [16] for a description) between both male types, as cave mollies may answer aggressions by sexually motivated behaviours [68].

## 3. Results

### 3.1. Body Size Difference between Opponents and Male Aggressive Behaviour

**Figure 1:**
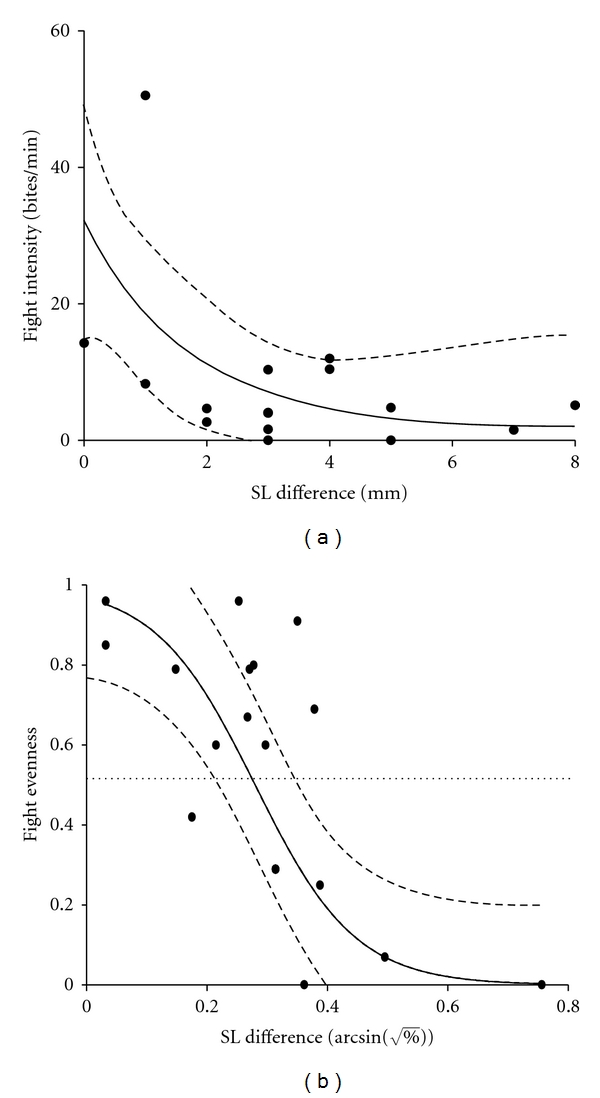
(a) Fight intensity and (b) “fight evenness” (see main text) in relation to the two opponents' body size difference (as standard length (SL) difference). Shown are regression lines representing the best-fit (a: logarithmic model; b: logistic model) and 95% confidence intervals (*N* = 17 fights).

In our first experiment we quantified fight intensities and durations in staged contests of *P. mexicana* males from the Tampico population. Body size differences between both males within a dyad varied between 0 and 8 mm. Fight intensity (measured as bites per minute) was negatively correlated with the opponents' body size difference (Spearman rank order test; *r*_*s*_ = − 0.52, *P* = 0.033; Figure 1(a)) meaning that males fought most intensely when both opponents were closely size matched. The body size difference below which fights escalated (i.e., below which both males displayed equal numbers of aggressive behaviours; “fight evenness”) was determined as 7.7%, with the 95% confidence interval ranging between 5.1% and 12.2% (Figure 1(b); Logistic model: *R*² = 0.51, *F*_1,16_ = 15.79). Based on these results, we made an attempt to use closely size-matched male pairs in all subsequent experiments [mean (±SD) size difference = 5.4 ± 8.2%] and included arcsine- (square root-) transformed relative body size difference of each dyad as a covariate in all further analyses.

### 3.2. Evolution of Male Aggressiveness in the Cueva del Azufre System

**Figure 2:**
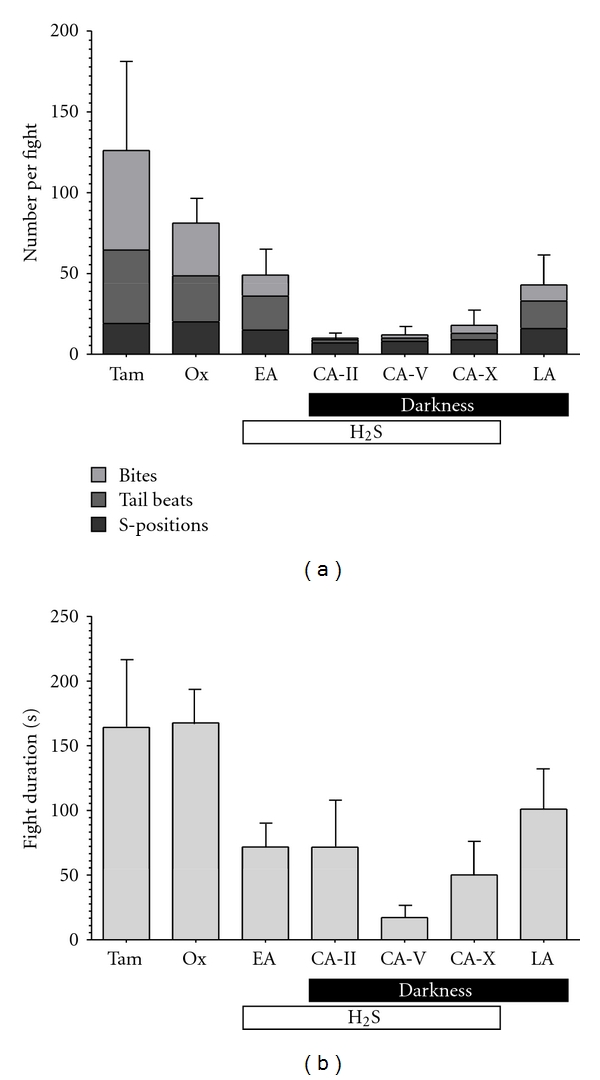
Means (±SE) of (a) numbers of aggressive interactions and (b) fight duration in seven populations of *P. mexicana. *From left to right: populations from nonsulphidic surface sites [Tampico, Tam (*N* = 12), and Río Oxolotán, Ox (*N* = 14)], the sulphidic creek in the Cueva del Azufre system [El Azufre, EA (*N* = 9)], three cave chambers of the sulphidic Cueva del Azufre [CA-II (*N* = 12), CA-V (*N* = 7), CA-X (*N* = 6)], and the H_2_S-free cave [Luna Azufre, LA (*N* = 10)].

MANCOVA revealed a significant effect of the factor “light” (Table 2(a)), indicating that cave-dwelling populations displayed significantly fewer aggressive behaviours than surface fish (Figure 2(a)). The significant “light × sulphide” interaction (Table 2(a)) further indicates that this reduction in aggressiveness is aggravated in populations evolving under both extreme conditions, while “sulphur” *per se* did not lead to a significant reduction in aggressive behaviours (Table 2(a); Figure 2(a)). Also both covariates (“mean opponent body size” and “body size difference”) had a significant influence in our model (Table 2(a)), and *post hoc* Spearman rank-order tests revealed that “mean opponent body size” was positively correlated with numbers of S-positions (*r*_*s*_ = 0.32, *P* = 0.007), tail beats (*r*_*s*_ = 0.30; *P* = 0.013), and bites per fight (*r*_*s*_ = 0.44; *P* = 0.001), suggesting that fights of larger males were more intense than those of smaller ones. In contrast, the body size difference between both opponents was negatively correlated with the number of S-positions (*r*_*s*_ = − 0.34; *P* = 0.004) and tail beats (*r*_*s*_ = − 0.24; *P* = 0.043), but not fights (*r*_*s*_ = − 0.19; *P* = 0.12), indicating that the larger the opponents' body size difference was, the less intense fights became.

When comparing the duration of fights we found both main factors (“light” and “sulphide”) to have significant effects (Table 2(b)). This and the nonsignificant interaction term of “light × sulphide” suggest that both the absence of light and the presence of H_2_S lead to similar reductions in fighting time (Figure 2(b)).

### 3.3. Aggressiveness in the Sulphur Endemic *P. sulphuraria*

**Figure 3:**
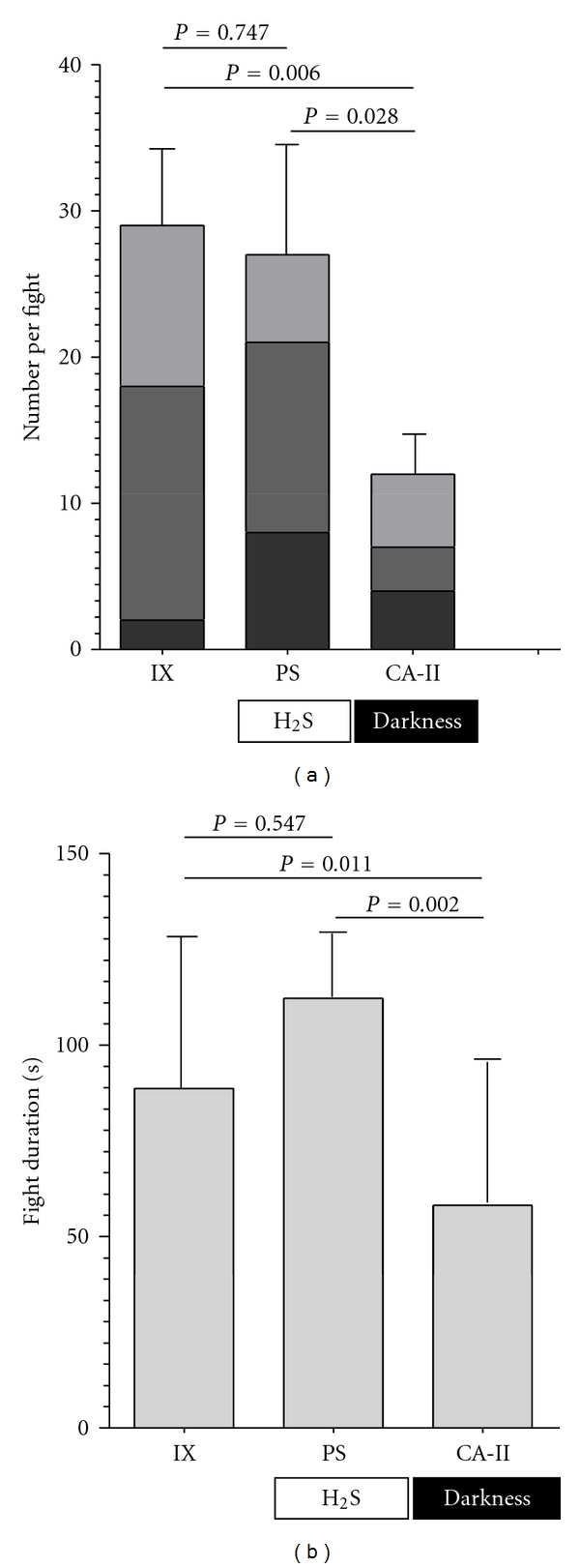
Means (±SE) of (a) numbers of aggressive behaviours shown by males during dyadic fights and (b) duration of fights in wild-caught males from two *P. mexicana *populations [the sulphide-free Río Ixtapangajoya, IX (*N* = 9) and cave chamber II of the sulphidic Cueva del Azufre, CA-II (*N* = 7)], as well as the sulphur-endemic *P. sulphuraria* (PS) found at the Baños del Azufre (*N* = 7).

When comparing numbers of aggressive behaviours in fights of wild-caught males from two *P. mexicana* populations (IX and CA-II) and *P. sulphuraria* males by use of MANCOVA we found a significant effect of the factor “population/species” (*F*_6,32_ = 3.54; *P* = 0.009), and *post hoc* pairwise comparisons (Fisher's LSD) showed levels of aggressive behaviours of surface *P. mexicana* and *P. sulphuraria* to differ significantly from those seen in *P. mexicana* males from CA-II (Figure 3(a)). None of the covariates had a significant effect (male body size difference: *F*_3,32_ = 1.24; *P* = 0.322; mean pair body size: *F*_3,32_ = 0.44; *P* = 0.722). One-way ANOVAs confirmed significant differences between populations in all three aggressive behaviours (S-position: *F*_2,20_ = 4.28, *P* = 0.028; tail beats: *F*_2,20_ = 7.51, *P* = 0.004; bites: *F*_2,20_ = 10.98, *P* = 0.001). *Post hoc* tests revealed that fights between *P. sulphuraria* males were characterized by significantly more S-positions compared to fights of IX males (*P* = 0.008), and fights of both surface populations/species displayed significantly more tail beats compared to fights of CA-II males (IX versus CA-II: *P* = 0.001; PS versus CA-II: *P* = 0.007). All three populations differed significantly in numbers of bites per fight (IX versus CA-II: *P* < 0.001; IX versus PS: *P* = 0.045; PS versus CA-II: *P* = 0.011).

When analysing the durations of fights, our ANCOVA model detected a significant effect of the factor “population/species” (*F*_2,18_ = 5.59; *P* = 0.013), and pairwise comparisons showed that both surface forms (IX and PS) fought significantly longer than *P. mexicana* males from CA-II (Figure 3(b)). Again, both covariates were not significant (male body size difference: *F*_1,18_ = 0.09; *P* = 0.763; mean pair body size: *F*_1,18_ = 0.02; *P* = 0.886).

### 3.4. Aggressiveness and Food Limitation

**Figure 4:**
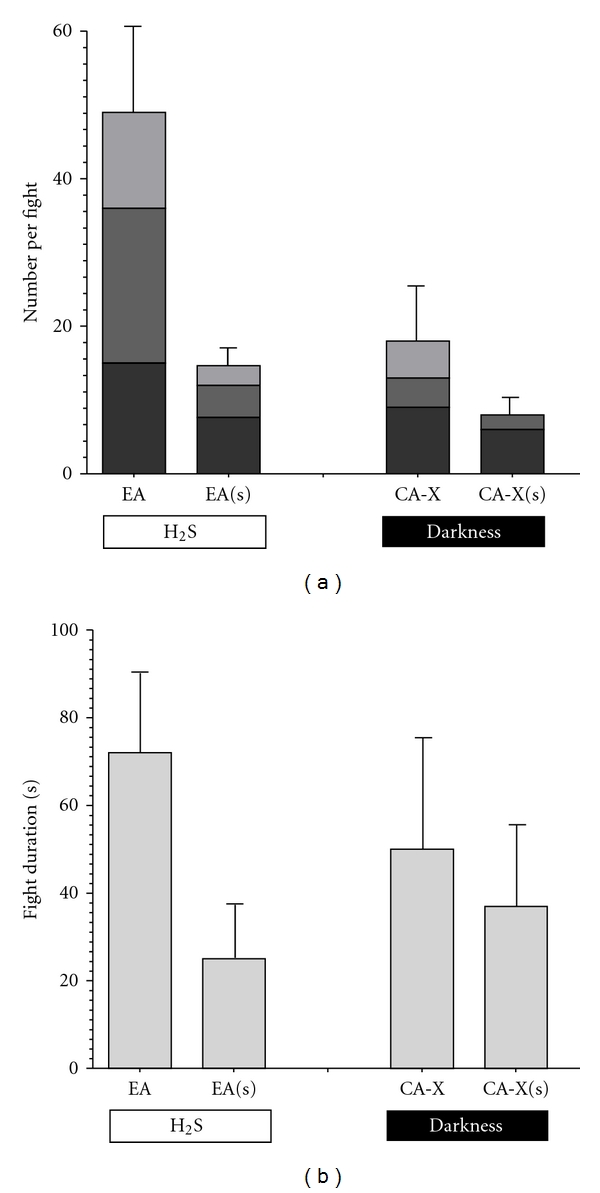
Means (±SE) of (a) numbers of aggressive behaviours and (b) the duration of male fights in males from two extremophile populations in the Cueva del Azufre system, one from the sulphidic surface stream [El Azufre, EA (*N* = 21)] and one from chamber X of the sulphidic Cueva del Azufre [CA-X (*N* = 16)]. Prior to the tests, males were either fed on a normal diet (left bars) or starved for one week (s: right bars).

When comparing numbers of aggressive behaviours in fights of *P. mexicana* from CA-X and EA under normal food supply (data from Experiment  2) and after one week of starvation in a MANCOVA we found a significant effect of the factor “food treatment” (*F*_3,29_ = 3.68; *P* = 0.023) while the factor “population” (*F*_3,29_ = 1.31; *P* = 0.29) as well as the interaction term “treatment × population” was not significant (*F*_3,29_ = 0.48; *P* = 0.70). This indicates that both populations reduced their aggressive behaviour in a similar fashion when food was scarce (Figure 4(a)).

Like in experiment  2, we found the covariate “mean opponent size” to have a significant effect in the MANCOVA (*F*_3,29_ = 3.28; *P* = 0.035), and *post hoc* Spearman rank-order tests revealed a significant positive correlation between “mean opponent size” and numbers of S-positions (*r*_*s*_ = 0.46; *P* = 0.004) and tail beats (*r*_*s*_ = 0.43; *P* = 0.007), but not bites (*r*_*s*_ = 0.35; *P* = 0.13). The covariate “body size difference” had no significant effect (*F*_3,29_ = 1.28; *P* = 0.30).

Another ANCOVA model analysing fighting durations revealed a significant effect of the factor “food treatment” (*F*_1,31_ = 4.44; *P* = 0.043) while the factor “population” (*F*_1,31_ = 0.02; *P* = 0.90) and the interaction term “treatment × population” were not significant (*F*_1,31_ = 1.76; *P* = 0.19). Furthermore, both covariates had no significant effects (“body size difference”: *F*_1,31_ = 1.10; *P* = 0.30; “mean opponent size”: *F*_1,31_ = 2.51; *P* = 0.12). Starvation in general reduced the duration of fights (Figure 4(b)).

### 3.5. Fights between Different Locally Adapted Males

**Figure 5:**
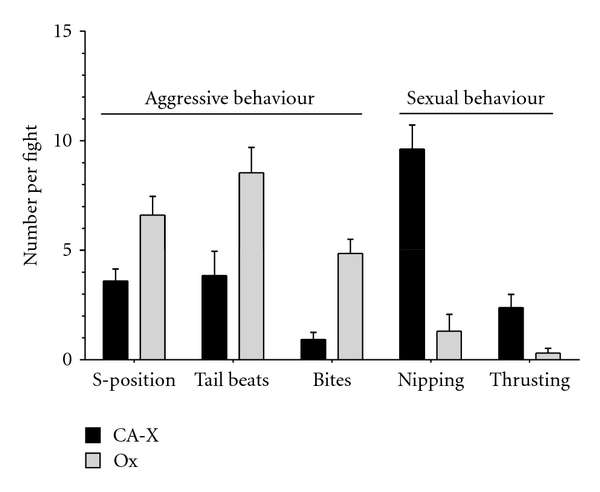
Mean (±SE) numbers of aggressive and sexual behaviours shown by males in cross-population dyadic fights (**N** = 13) between Río Oxolotán (Ox) males and males from chamber X of the sulphidic Cueva del Azufre (CA-X). All comparisons were statistically different between populations (see main text).

In all 13 staged contests, Río Oxolotán (Ox) males established dominance over the CA-X males (Chi^2^ = 13.0, df = 1, *P* < 0.01) after a mean fight duration of 119 ± 19 s. Ox males directed significantly more aggressive behaviours towards CA-X males (S-position: *t*_12_ = −4.12, *P* = 0.001; tail-beats: *t*_12_ = − 4.50, *P* < 0.001; bites: *t*_12_ = − 5.38, *P* < 0.001; Figure 5) while cave molly males directed more sexually motivated behaviours towards Ox males during the fights (nipping: *t*_12_ = 4.49, *P* < 0.001; thrusting: *t*_12_ = 3.43, *P* = 0.005; Figure 5).

## 4. Discussion

An increasing body of literature documents adaptation's potential to drive genetic differentiation and ultimately speciation (e.g., [80–83]), a phenomenon that has recently been termed “isolation by adaptation” [84]. Of particular interest in the study of ecological speciation are the proximate mechanisms leading to and maintaining genetic differentiation among populations [2, 84]. During ecological speciation, prezygotic isolation may arise when immigrants from foreign, ecologically divergent habitats are selected against [85, 86]. This may occur through natural selection, if immigrants (or hybrids) have reduced viability (extrinsic reproductive isolation; e.g., [5, 6, 87]), or by sexual selection, if maladapted individuals are discriminated against during mate choice (e.g., [88]).

In the present study we examined whether—in addition to mate choice (i.e., intersexual selection)—intrasexual selection through male-male competition could also play a role in promoting prezygotic isolation. Atlantic molly males in clear-water habitats usually establish dominance hierarchies, and dominant (typically the largest) males monopolize several females which they aggressively defend against rivals [16]. This view is supported by our present findings in that fighting intensity was positively correlated with the average body size of male dyads; in other words, larger males fought more intensely, probably driven by the prospect of monopolizing females. Smaller males, by contrast, rely on a sneak-like mating tactic [50, 70], but such “alternative” mating tactics are lost in extremophile *P. mexicana *[50], likely owing to very similar counterselection in energy-limited habitats that, as we will discuss, may have played a role for the evolutionary reduction of aggressive behaviour (see below).

We found fight intensity to be reduced in various extremophile *P. mexicana* populations, and perpetual darkness in caves was the best predictor for the evolutionary reduction of aggressiveness, especially when it was combined with presence of H_2_S, as seen in the CA cave. As lab-reared fish were used for this part of our study, the observed differences seem to be largely evolved (genetic) differences among ecotypes. When we considered fight durations, also a significant main effect of the factor “H_2_S” was observed; fish from sulphidic habitats engage in shorter fights as an evolutionary response to the toxicant. Finally, we demonstrate that reduced aggression directly translates into males being inferior in contests, as evidenced by the fact that Ox males always won when paired with cave molly (CA) males; CA males even responded sexually to aggressive attacks, an obviously maladaptive behaviour (see also [68]). We argue that in a system where dominance hierarchies play a vital role, reduced aggressiveness translates directly into male inferiority in mate competition upon encounter of different behavioural phenotypes. Specifically, we argue that migrant males stemming from an ecological background that has selected for reduced aggressiveness may be selected against (i.e., have low reproductive fitness) in a divergent (i.e., benign) habitat type. Together with the action of natural selection against (maladapted) migrants via H_2_S-toxicity, darkness and predation [5, 6, 8, 26, 59], as well as female mate discrimination against alien male phenotypes [5, 8], divergent evolution of aggressive behaviour may thus play an important role for the maintenance of genetic differentiation in this system—at least at the interface between extreme and benign (nonsulphidic surface) habitats and, hence, could represent another mechanistic link explaining the surprising small-scale genetic structuring in the CA system [41, 42]. It remains to be determined in future studies whether the comparatively small differences in the intensity of aggressive behaviour seen in males from some habitats that are directly adjoining in the Cueva del Azufre system (e.g., CA versus EA, LA versus EA) lead to an equally clearcut picture, that is, if also in those cases it is always the more aggressive males that win a combat. For practical reasons, our present study focussed on aggressive interactions between the most extreme behavioural phenotypes: males from the most aggressive (Ox) population and the least aggressive CA-X males.

Parzefall first described reduced aggression in the CA cave population of *P. mexicana* (from the rearmost chamber XIII; CA-XIII) [16] and interpreted his findings as an adaptation to perpetual lightless conditions [17], as most aggressive behaviours depend on visual perception of cues from opponents, which may be more difficult to perceive in darkness. Even though theory predicts a reduction of intraspecific aggression in troglobites [89, 90], some cave dwellers may even have evolved entire novel sets of aggressive behaviours while responding to nonvisual signals. For example, aggressive behaviour is well developed in the blind catfish *Uegitglanis zammaranoi* [91] and the blind cave salamander *Proteus anguinus* [92]. Furthermore, the discovery of a highly aggressive cave-dwelling *Astyanax fasciatus* population [93] implies that a reduction of aggressive behaviour is not an inevitable evolutionary response to the cave environment. Those authors suggested that explanations other than simply the inability to perceive visual cues triggering aggressive behaviour should be explored in order to explain the evolutionary reduction of aggressiveness in many other *Astyanax* cave fish populations [94, 95].

As we have argued above, previous studies have demonstrated that *P. mexicana* inside the two caves (CA and LA), as well as the toxic surface habitat (EA), appear to be energy limited, as evidenced by their lower body conditions and reduced fat stores [27–30, 52]. In the nontoxic LA cave, this is probably due to low resource availability, which is typical for most caves (reviewed in [37, 96]). In contrast, CA and EA are energy-rich habitats due to high chemoautotrophic primary productivity [97, 98]; however, *P. mexicana* spend the majority of their time at the water surface engaged in ASR [26] and probably pay a high physiological cost in order to run ATP-expensive H_2_S detoxification [22]. Not surprisingly, a recent study therefore found cave mollies from CA to have higher metabolic rates compared to surface mollies even after several generations in the laboratory [99]. Altogether, this suggests that reduced aggression is most likely an evolutionary response to continued energy limitation in the Cueva del Azufre system. This hypothesis is corroborated by the results from this study, in which we found that *P. mexicana* from EA and CA plastically reduce their aggression even further after being starved for one week.

It is further interesting to note that not only did overall levels of aggression diverge between extremophile and nonextremophile poeciliids, but also the relative contribution of specific aggressive behaviours to the aggressive repertoire of these species. The potentially most harmful aggressive behaviour (i.e., bites and rammings) was strongly reduced in all extremophile poeciliids, while the least harmful behaviour (i.e., S-positions) was actually increased in extremophiles. As the energetic costs of threat displays were found to be low relative to the costs of escalated fighting in an African cichlid species (*Tilapia zillii*) [100] we argue that again this phenomenon is a response to the energy limitation experienced in extreme habitats. Moreover, *P. mexicana* males appear to have higher energy expenditure than females [25, 101] and, therefore, exhibit higher mortality rates under stressful conditions and perform more ASR than females [25]. Assuming that male poeciliids in H_2_S-toxic habitats live near the edge of survivability [25], any injuries obtained during fights with other males (especially during biting or ramming) could indeed lead to life-threatening infections and, ultimately, premature death—a hypothesis that is further supported by a recent study reporting on higher individual parasitization rates of *P. mexicana* in the CA and EA compared to Ox [102].

In stark contrast to the findings from the Cueva del Azufre system, our experiments using wild-caught males from another system with high and sustained H_2_S, namely, *P. sulphuraria* inhabiting the Baños del Azufre, found no significant difference among *P. mexicana* from nonsulphidic sites and the “sulphur-endemic” *P. sulphuraria*. So, why did extremophile males from the Cueva del Azufre system show strongly reduced aggressiveness, but *P. sulphuraria* did not? We propose three mutuallynot exclusive hypotheses. First, our analysis of fish in the Cueva del Azufre system found the relative contribution of the factor “sulphide” to the evolutionary reduction of aggressive behaviour to be much lower than that of the factor “light” (see partial variance explained in Table 2), so these fish may just not experience the same selective pressure to reduce aggressiveness. Second, *P. sulphuraria* are clearly well adapted to high concentrations of H_2_S (i.e., being sulphur endemics) and accordingly could show some kind of “rebound effect”, indicating evolved mechanisms to better cope with the toxicant (see [20] for discussion). Some support for the latter idea was also found in life-history traits, as *P. sulphuraria* actually had the largest (not the smallest) fat stores in a comparison of poeciliids from several benign and sulphidic habitats [27]. Third, contrary to *P. mexicana* from the Cueva del Azufre system, which are the only permanent piscine residents in their respective extreme habitats [43], *P. sulphuraria* have to share their habitat with another sulphide-adapted species, the widemouth gambusia, *Gambusia eurystoma* [40]. Hence, increased aggression could also be a signal of interspecific competition for resources at the Baños del Azufre.

In conclusion, sulphuric waters are characterized by reduced resource availability but increased energy expenditure, leading to low body conditions and fat stores in H_2_S-inhabiting *P. mexicana*. We suggest that in addition to darkness in caves also resource limitation might play a crucial role in the evolutionary reduction of male aggressive behaviour. Selection against costly behaviours (such as aggression) might lead sulphur-adapted mollies to trade-off aggressive behaviour to compensate for the negative effects of H_2_S—similar to the proposed processes resulting in the observed heritable reduction of male sexual activity and harassment of females found in all extremophile populations (e.g., [50]), as well as patterns of life history divergence [28, 29]. On the other hand, the phylogenetically old “sulphur specialist” *P. sulphuraria*, which we did not find to show reduced aggression, might cope better with H_2_S and thus can afford to express costly aggressive behaviour.

## Figures and Tables

**Table 1:** Overview of populations used in this study. Given are relevant ecological habitat parameters [light absent (−) or present (+); H_2_S absent (−) or present (+)], origin of test individuals [lab-reared (lr) or wild-caught (wc)], as well as coordinates of the sampling sites.

	Light	Sulphide	Origin	Latitude	Longitude
Tampico (Tam)	+	−	lr	22.29632	−97.90022
Río Oxolotán (Ox)	+	−	lr	17.44444	−92.76293
El Azufre (EA)	+	+	lr	17.44225	−92.77447
Cueva del Azufre II (CA-II)	−	+	lr	17.44225	−92.77447
Cueva del Azufre V (CA-V)	−	+	lr	17.44225	−92.77447
Cueva del Azufre X (CA-X)	−	+	lr	17.44225	−92.77447
Cueva Luna Azufre (LA)	−	−	lr	17.44225	−92.77447
Río Ixtapangajoya (IX)	+	−	wc	17.49450	−92.99763
*Poecilia sulphuraria* (PS)	+	+	wc	17.55225	−92.99859
Cueva del Azufre II (CA-II)	−	+	wc	17.44225	−92.77447

**Table 2:** Results from (a) MANCOVA and (b) ANCOVA models analysing attributes of dyadic male aggressive interactions in Experiment  2 (lab-reared males). *F*-ratios were approximated using Wilk's *λ*. Partial variance was estimated using Wilk's partial *η* ^2^. Significant effects are in bold typeface.

	df	*F*	*P*	Partial variance explained [%]
(a) MANCOVA (number of aggressive behaviours)				
Light (absent/present)	**3,62**	**8.97**	**<0.001**	**0.30**
Sulphide (absent/present)	3,62	2.45	0.072	0.10
Light × sulphide	**3,62**	**3.77**	**0.015**	**0.15**
Male body size difference	**3,62**	**3.35**	**0.025**	**0.14**
Mean pair body size	**3,62**	**2.81**	**0.044**	**0.12**

(b) ANCOVA (fight duration)				
Light (absent/present)	**1**	**8.44**	**0.005**	**0.12**
Sulphide (absent/present)	**1**	**7.07**	**0.010**	**0.10**
Light × sulphide	1	2.48	0.120	0.04
Male body size difference	1	0.25	0.622	<0.01
Mean pair body size	1	0.71	0.403	0.01
Error	64			
